# Leveraging machine learning to uncover the hidden links between trusting behavior and biological markers

**DOI:** 10.1080/19585969.2025.2513697

**Published:** 2025-06-20

**Authors:** Zimu Cao, Daiki Setoyama, Monica Natsumi Daudelin, Toshio Matsushima, Yuichiro Yada, Motoki Watabe, Takatoshi Hikida, Takahiro A Kato, Honda Naoki

**Affiliations:** ^a^ Laboratory for Theoretical Biology, Graduate School of Biostudies, Kyoto University, Kyoto, Kyoto, Japan; ^b^ Department of Clinical Chemistry and Laboratory Medicine, Graduate School of Medical Sciences, Kyushu University, Fukuoka, Japan; ^c^ Department of Neuropsychiatry, Graduate School of Medical Sciences, Kyushu University, Fukuoka, Japan; ^d^ Laboratory for Advanced Brain Functions, Institute for Protein Research, Osaka University, Osaka, Japan; ^e^ Laboratory for Data-driven biology, Graduate School of Medicine, Nagoya University, Nagoya Aichi, Japan; ^f^ Sunway Business School, Sunway University, Petaling Jaya, Malaysia; ^g^ Department of Psychiatry, Hokkaido University Graduate School of Medicine, Sapporo, Japan; ^h^ Laboratory for Data-driven Biology, Graduate School of Integrated Sciences for Life, Hiroshima University, Higashihiroshima, Hiroshima, Japan; ^i^ Theoretical Biology Research Group, Exploratory Research Center on Life and Living Systems (ExCELLS), National Institutes of Natural Sciences, Okazaki, Aichi, Japan

**Keywords:** Machine learning, decision making, biomarker

## Abstract

Understanding the decision-making mechanisms underlying trust is essential, particularly for individuals with mental disorders who often experience difficulties in forming interpersonal trust. In this study, we aimed to explore biomarkers associated with trust-based decision-making through quantitative analysis. However, quantifying internal decision-making processes is challenging, as they are not directly observable. To address this, we developed a machine learning method based on a Bayesian hierarchical model to quantitatively infer latent decision-making parameters from behavioural data collected during a trust game. Applying this method to data from patients with major depressive disorder (MDD) and healthy controls (HCs), we estimated individualised model parameters that regulate trust-related decisions. The model successfully predicted participants’ behaviours in the task. Although no significant group-level differences were observed in the estimated parameters between the MDD and HC groups, we uncovered hidden links between trust-related decision-making processes and specific blood biomarkers. Notably, metabolites such as 5-aminolevulinic acid, acetylcarnitine, and 2-aminobutyric acid were significantly associated with individual differences in trusting behaviour. These findings provide valuable insight into the biological basis of trust-based decision-making. They also offer a novel framework for integrating behavioural modelling with biomarker discovery, potentially informing the development of targeted interventions to enhance social functioning and overall well-being.

## SUMMARY

Our society is founded on trust. At the individual level, we make decisions by assessing the other person’s trustworthiness, and disturbances in trustworthiness are associated with various psychiatric disorders. Therefore, deciphering decision-making abnormalities from blood data is an important issue in the field of psychiatry. However, research to date has been limited to identifying biomarkers that predict directly observable behaviours and health status, and methods to explore biomarkers that assess trust-based decision-making processes that cannot be directly observed has not fully been established. Here, we established a mathematical model for decision making in trust games and developed a machine learning method to quantitatively evaluate the decision-making process based on participants’ behavioural data. By comparing the estimated model parameters with blood data for each participant of the trust game, we discovered biomarkers that predict characteristics of trust-based decision making. Such an approach proposes a new concept of analysing blood data reflecting the immune and metabolic systems, in relation with decision making which has traditionally been difficult to quantify. Our study bridges the gap between psychiatry and biology, offering a fresh lens through which we can understand and address the complexities of human trust-based decision-making.

## Introduction

Humans engage in trusting behaviours that rely on their first impressions of others. For example, voting and cooperative activities such as business can be seen as trusting behaviours [1,2]. Patients with major depressive disorder (MDD) often face difficulties in developing trust, which can be interpreted as a defect in decision making [3–5]. Similarly, *hikikomori*—a severe form of pathological social withdrawal or social isolation that persists for more than six months and entails rarely leaving home—exhibit similar impairments in trust [3–5]. Notably, a portion of individuals with hikikomori are also diagnosed with MDD [6]. Many studies have only described statistical differences in trusting behaviours between patients with MDD and healthy controls (HCs) using standard psychology methods such as behavioural tasks and questionnaires [7,8]. Understanding decision-making mechanisms that depend on trust, which is latent and not directly observed, is important. However, to the best of our knowledge, no method has been established to decode the degree of trust in decision-making behaviours. Therefore, we developed a machine learning method to model trusting behaviours based on the first impression of others’ facial pictures using a trust game.

A trust game is an economic game used to assess the degree to which people trust others [9]. The trust game consists of two players, one of whom is asked to evaluate the level of trust in the other and then make a financial decision with a risk. The advantage of using the trust game is that it objectively obtains patients’ interactions, that is, the amount of money the patients pay, in contrast to the traditional diagnosis of psychiatric disorders, most of which are based on subjective clinical interviews. Several clinical studies using economic games have revealed that patients with major depression and personality disorders experience difficulties in social decision-making involving trust [10,11]. In our previous study using the trust game, we found that male patients with MDD exhibited more trusting behaviour towards females of higher attractiveness [3]; however, most of these studies only conducted descriptive statistical analyses of trust behaviours and did not explore the mechanisms underlying decision making during trust behaviours.

Meanwhile, some blood biomarkers have been shown to change in patients with psychiatric disorders. We previously investigated blood biomarkers in hikikomori patients, who avoided social interaction to an extreme extent due to their inability to build trusting relationships with others [6]. We found that these patients had decreased blood uric acid levels [12]. In addition, changes in blood biomarkers have been reported in patients with MDD, including decreased brain-derived neurotrophic factor (BDNF) levels and increased C-reactive protein (CRP) levels [13,14]. Furthermore, we demonstrated that minocycline modulates social behaviour, suggesting that blood chemical levels play an important role in trust-related behaviour [15]. However, the hidden links between biological markers and trust-related behaviours remain elusive.

To explore the biomarkers associated with trust behaviour, we proposed a classification scheme comprising three levels of biomarkers. The first level includes biomarkers that are predictive of latent mental states, such as healthy conditions or depression. These biomarkers—such as CRP, BDNF, and uric acid—have been well-studied in psychiatric research and are relatively easy to detect due to their association with diagnosed clinical states [12–14]. The second level comprises biomarkers, such as the minocycline, that are linked to directly observable behavioural outcomes, such as investment decisions in social interaction tasks or responses in trust-related paradigms [15]. The third level reflects latent decision-making processes (DMPs), which involve internal computations transforming environmental inputs (e.g., facial cues or social signals) into behavioural outputs. While first- and second-level biomarkers are straightforward to assess, DMPs are not directly observable and must be inferred using computational models. Consequently, third-level biomarkers remain poorly understood despite their potential importance in capturing the mechanisms underlying trust behaviour.

This study aimed to uncover hidden links by identifying third-level biomarkers associated with latent DMPs. The strategy is as follows: First, we developed a machine learning method to quantitatively decode the DMP in the trust behaviours of both patients with MDD and HCs. This method was based on a Bayesian hierarchical model that describes the decision-making processes in a trust game. Using the MDD and HC data, we estimated the model parameters, which regulated trust behaviour. In addition, we quantified the characteristics of trusting behaviour in patients with MDD and identified several biomarkers associated with the properties of the MDP involved in trust behaviour.

## Results

### Trust game and blood biomarkers

**Figure 1.**
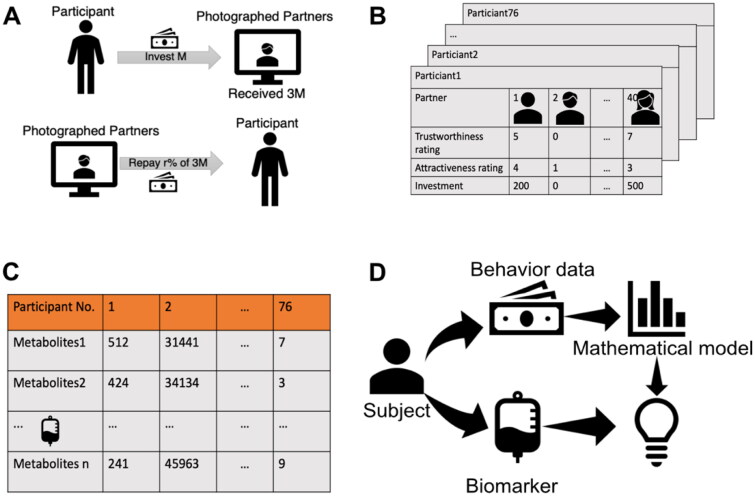
Trust game and blood biomarker measurement. **(A)** Trust game with the photographed partners. A participant is asked to rate the trustworthiness and attractiveness of the partner in the display and to invest 0–1300 yen. **(B)** Behavioural data for the trust game. 38 healthy controls and 38 patients with MDD participated in the trust game. Each participant plays the trust game with 40 photographed partners. **(C)** Measurement of blood biomarkers. Eighty-five metabolites were measured for all participants. The three panels depict examples of metabolite measurement, which show statistically significant differences between HCs and patients with MDD. **(D)** Scheme of this research. For all participants, behavioural data from the trust game and blood biomarker data of 85 metabolites are obtained. From the behavioural data, participant-specific parameters of the decision-making model are estimated. By integrating the estimated parameters and blood biomarker data, we identified the molecular basis of trusting decision making.

To investigate the relationship between biomarkers and decision-making processes in trust behaviours in patients with MDD, we performed psychological tasks and collected blood biomarkers. Trusting behaviour was examined using a trust game (Figure 1A). The trust game we adopted included two players: participants of interest and photographed partners on the PC screen. All participants were asked to score the trustworthiness and attractiveness of the partner using facial information and then make a financial decision on whether to give them some amount of money (Figure 1B). Before playing the game, the participants were initially given some funds and told that their partner had obtained three times the money they were given and would possibly return a portion to them. In addition, all participants provided a non-fasting venous blood sample from which we measured 85 biomarkers (Figure 1C). For comparison, we performed the experiments on patients with MDD and HCs.

### Several types of biomarkers

**Figure 2.**
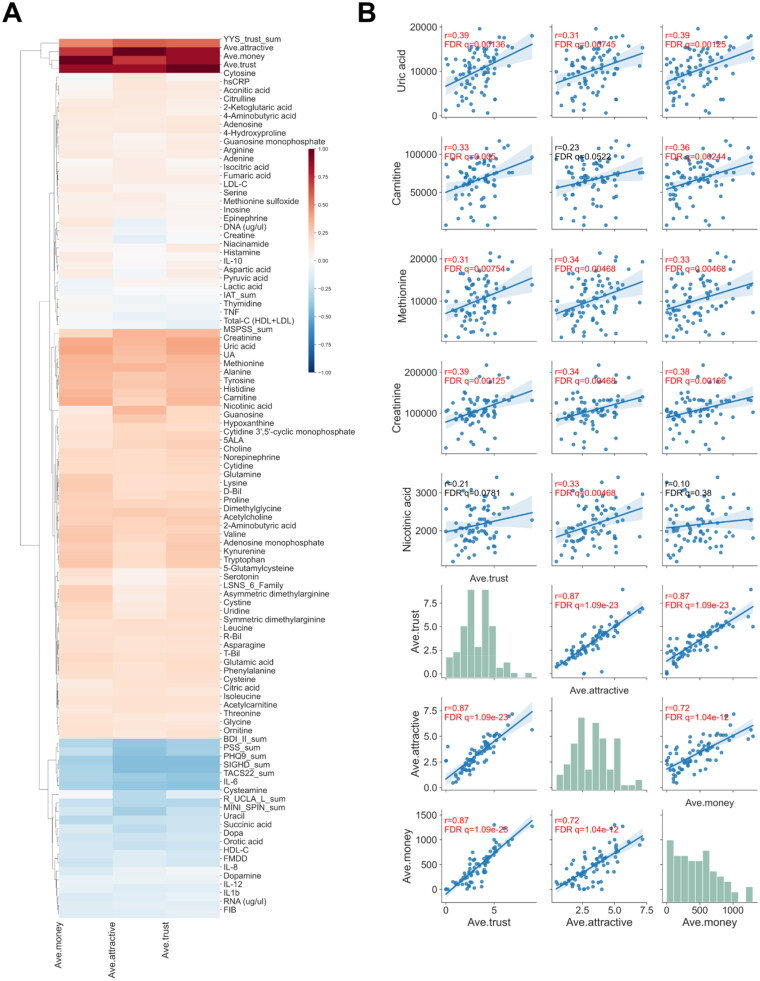
Blood biomarkers correlated with behaviours in the trust game. **(A)** Heatmap of correlation coefficients between the all-measured biomarkers and three kinds of behaviours. Hierarchical clustering was performed. Spearman correlation was conducted for correlation analysis. **(B)** Scatter plot of parameters and biomarkers for selected pairs. Correlation coefficients with an FDR-corrected q-value < 0.05 were considered significant, which were coloured by red. The prefix “Ave”, indicates the average value across all face stimuli for each participant (e.g., average evaluation or investment amount across all partners).

Many researchers have aimed to identify biomarkers that can predict diseases and healthy states. Typically, biomarkers have been considered to predict whether an individual is healthy or in a state of MDD. Previously, we used machine learning to identify biomarkers by classifying healthy controls (HCs) and individuals with Hikikomori [6], among whom 24% (10/42) were also diagnosed with MDD. In this study, we found that patients with MDD showed higher concentrations of orotic acid and lactic acid, and lower concentrations of cystine, arginine, and methionine in the blood compared to HCs (Supplementary Fig. 1). Another type of biomarkers is indicators that directly predict the participant’s answers and behaviours in the task, which can be identified by a native comparison between behavioural and blood sample data. In fact, we found that participants with higher concentrations of uric acid, carnitine, methionine, creatinine, and nicotinic acid scored higher on the trustworthiness and attractiveness of the photographed partners and invested more money to their partners (Figure 2). However, it remains to be elucidated how these two types of biomarkers are related to the decision-making process in trust behaviours.

In this study, we aimed to identify biomarkers reflecting the decision-making process, that is, the transformation from evaluation of the partner (attractiveness and trustworthiness) to payment behaviours. To this end, we needed to model the decision-making process and estimate the model parameters that reflect the decision-making properties for each participant. The strategy of this study was to integrate data from the trust game and the biomarkers (Figure 1D).

### Bayesian hierarchical model for trust-based investment

**Figure 3.**
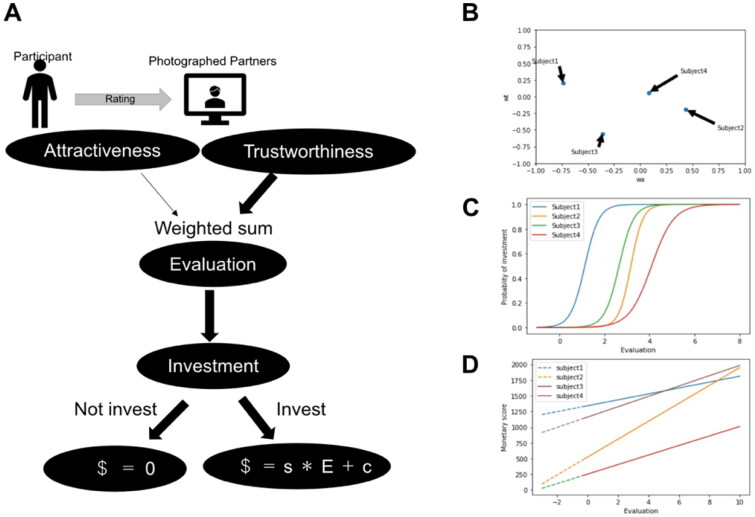
Decision-making model in trust game. **(A)** Decision-making process towards investment. The participants’ ratings for the photographed partners, investments, and monetary scores are observed, whereas the model parameters are unknown. **(B)** Weights for attributes of photographed partners are participant specific. **(C)** Decision of whether to make an investment. Participants tend to make an investment for highly evaluated photographed partners. The probability of investment obeys subject-specific sigmoid function. **(D)** Decision of the investment amount. If the participant decides to invest, his/her monetary score to invest linearly increases with the evaluation of the photographed partners in a participant-specific manner. Otherwise, the monetary score is zero.

We developed a Bayesian hierarchical model to capture the decision-making process involved in trust-based investments (Figure 3A). Specifically, our model considers the evaluation of a partner’s face to be a crucial determinant of investment decisions. In the model, the participant evaluated the partner *j* as

$$E_{j}=w_{o}+w_{t}\;t_{j}+w_{a}\;a_{j}+\text{noise}$$

where $t_{j}$ and $a_{j}$ indicate the ratings of trustworthiness and attractiveness of the *j*-th photographed partner, respectively; $w_{o}$, $w_{a}$, and $w_{t}$ indicate the weights of the basal evaluation, attractiveness, and trustworthiness, respectively. The participant decides whether to invest, based on the following probability:

$$\mu (E_{j})=\frac{1}{\left(1+\text{exp}(-\beta (E_{j}-\gamma ))\right)}$$

where β and γ indicate constant parameters, which control randomness and threshold of investment, respectively. If a decision to invest is made, the amount of money invested is determined as

$$m=s\;E_{j}+c+\text{noise}$$

where s and c are constant parameters. If a decision to not invest is made, then the amount of money invested is zero. Furthermore, we assume that all parameters (i.e., $w_{o}$, $w_{a}$, and $w_{t}$) are participant specific (Figure 3B-D) and sampled from prior distributions (see Methods for details). Summarily, each participant in our model has their own weights for trustworthiness and attractiveness in the evaluation of partners’ faces. After evaluating their partner’s face, the participant decides whether to invest based on the weighted evaluation. If they decide to invest, a monetary score is determined through a weighted evaluation.

### Biased evaluation by patients with MDD

**Figure 4.**
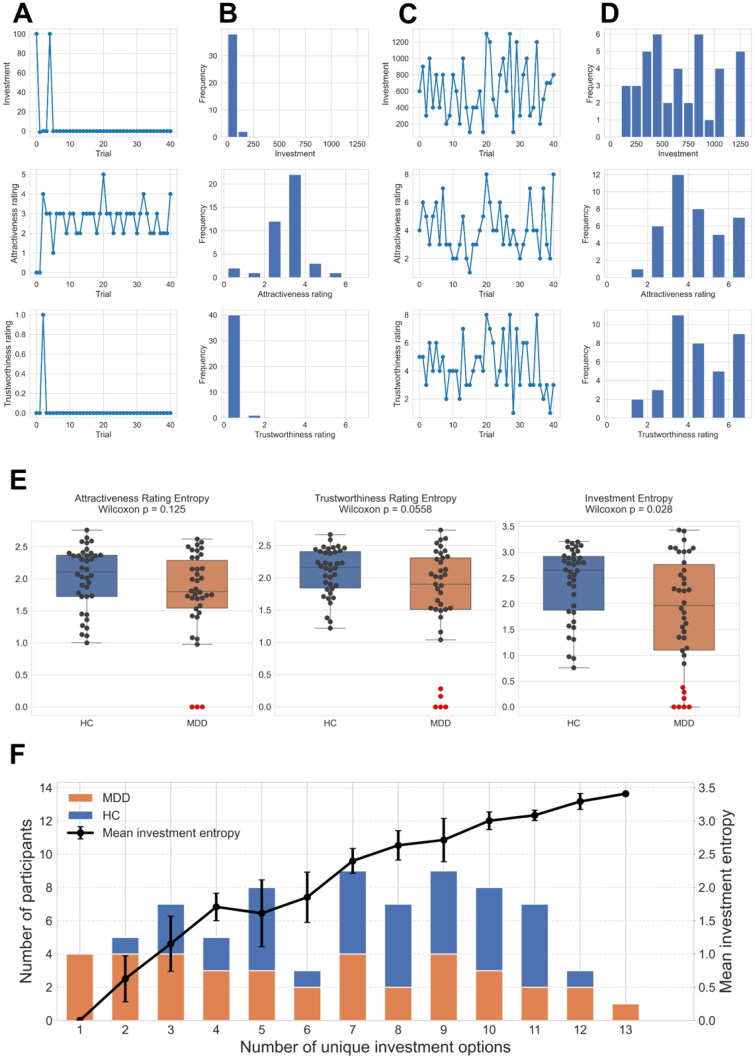
Biased, non-serious behaviours in trusting game. **(A)** Representative participants showing non-serious responses of attractiveness/trustworthiness ratings and monetary score of investment. **(B)** Histogram of each response for the representative participants in (A). **(C)** Representative participants showing diverse range of choices of attractiveness/trustworthiness ratings and monetary score of investment. **(D)** Histogram of each response for the representative participants in (C). **(E)** Comparison of Shannon entropy for each response between HCs and patients with MDD (Wilcoxon rank-sum test). Seriousness of participants for each response was quantified using Shannon entropy *S*. When outliers with entropy values below 0.5 were excluded, the statistical differences between groups disappeared (p-values = 0.3176, 0.3135, and 0.3437 for attractiveness rating, trustworthiness rating, and monetary investment, respectively). **(F)** Histogram (left y-axis) of the number of participants who used *k* distinct investment amounts (0–1 300 JPY in 100-JPY steps, 14 options in total). Orange bars: MDD; blue bars: HC. The black line (right y-axis) depicts the mean Shannon investment-entropy for participants with each option count; error bars denote ± SD.

Before applying the Bayesian hierarchical model to the real data of the trust game, we conducted a native comparison of behavioural responses between HCs and patients with MDD and found that MDD group showed significantly lower investment amounts, attractiveness ratings, and trustworthiness ratings compared to HCs (Supplementary Fig. 2). To further characterise how each participant evaluated different partners, we conducted a more detailed analysis of individual response patterns. In particular, we noticed that some participants did not take the game seriously, with biased face evaluations and/or investments. Some participants always made the same evaluation for different partners’ faces (Figure 4A and B) and invested the same amount of money in all partners. Conversely, other participants exhibited a more diverse range of choices (Figure 4 B and C). In traditional psychological surveys, biased answers such as choosing only one or few options out of many according to subjective criteria are considered invalid. Thus, we quantified such biased answers for each participant using Shannon entropy [16], which is used in information theory to measure the variability of variables; high and low Shannon entropies represent varied and biased answers, respectively.

We found that patients with MDD displayed lower entropy in both their investment amounts and their trustworthiness ratings when compared with healthy controls (Wilcoxon rank-sum: *p* = 0.028 and *p* = 0.055, respectively; Figure 4E). To assess what level of entropy reflects meaningful engagement with the task, we created a histogram showing the number of distinct investment amounts selected by each participant and calculated the corresponding mean entropy for each bin. This analysis revealed that participants who selected three or less distinct options had an average investment entropy smaller than 1.5 (Figure 4F). Based on this finding, we determined that an entropy threshold of 1.5 provides a more appropriate criterion for excluding participants who did not fully engage with the task. Thus, we excluded responses with entropy < 1.5 as non-informative samples from subsequent analyses.

### Estimation of participant-specific parameters

**Figure 5.**
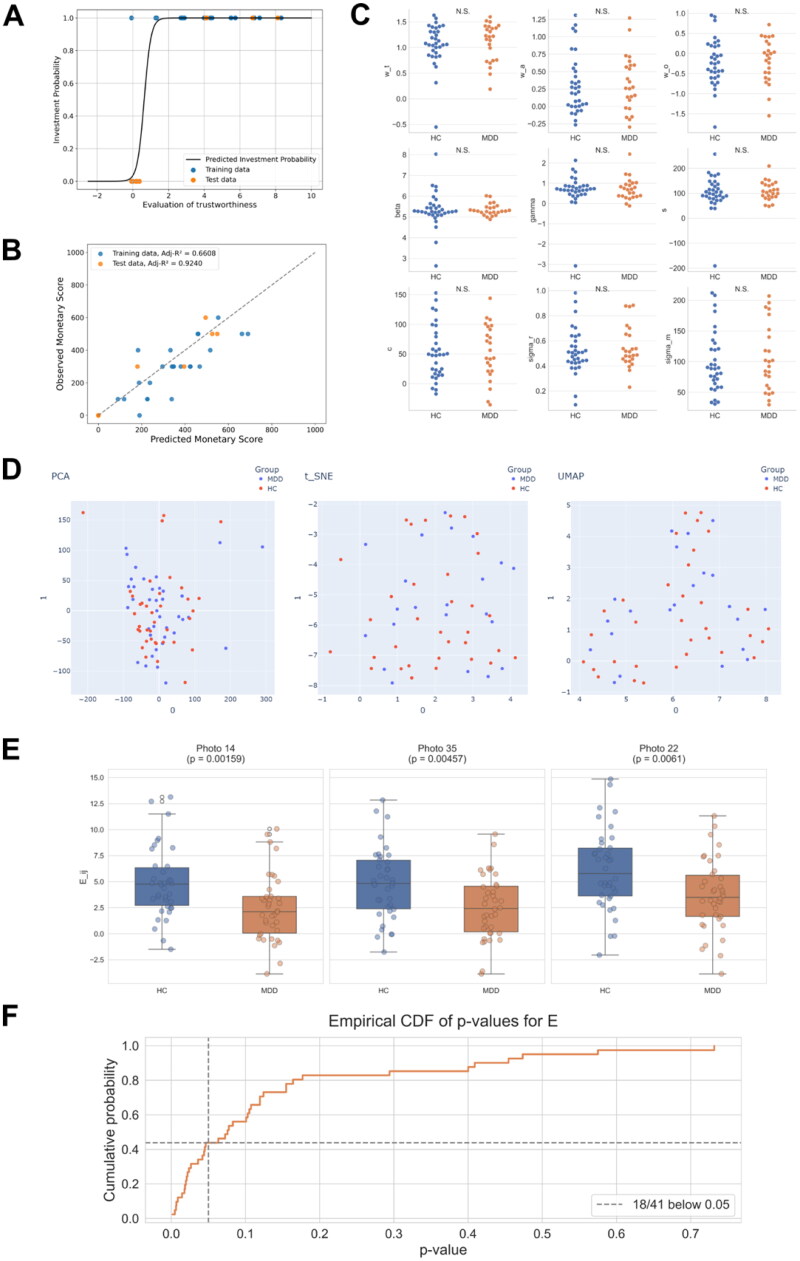
Prediction of participants’ behaviours in the trust game and parameter comparison between HCs and patients with MDD. **(A)** Predictions of whether each participant invests in the photographed partner. **(B)** Predictions of how much money each participant invests. **(C)** Comparison of the estimated parameters between HCs and patients with MDD. N.S. No significance. (Welch’s two-sample t-test). **(D)** Visualisation of participants in model parameter space in 2D space. PCA, t-SNE, and UMAP were performed for dimension reduction of the estimated parameters. **(E)** Comparison of model-derived evaluations Eij between HCs and patients with MDD. Each panel shows the distribution of Eij values for representative photographed partners. Group differences were tested with the Mann-Whitney U test (*p* < 0.05). **(F)** Cumulative distribution of U-test *p*-values for Eij across all 41 photographed partners. The dashed line marks *p* = 0.05; 18 faces fall below this threshold.

Based on the developed model, we aimed to determine the participant-specific parameters from the trusting behaviours (i.e., the participants’ scores of trustworthiness and attractiveness of their partners and investment amount) in the trust game. Using the MCMC method, we obtained MAP point estimates for all parameters from the training data (see Methods). To evaluate the convergence of the MCMC sampling process, we additionally analysed the time-course of the log posterior values. This analysis revealed that the posterior values stabilised after the initial 2,000-step burn-in period, showing only minor fluctuations thereafter (Supplementary Fig. 3). We then demonstrated that the participant-specific models were able to predict whether and to what extent HCs and patients with MDD invested in their partners, which was confirmed by the test data (Figure 5A and B).

We then examined how trust behaviours differed between the HC and MDD groups. Here, we used parameters estimated from the data for all 41 partners, which should be more reliable than those estimated from the partial training data. We found no significant differences in any of the nine parameters between the groups (Figure 5C). In addition, we performed dimension reduction analyses (e.g., PCA, T-SNE, and UMAP) on a set of nine parameters, which characterised participant-specific trust behaviours (Figure 5D). Furthermore, we also applied supervised classification methods, including Linear Discriminant Analysis (LDA) and logistic regression, to evaluate whether these parameters could separate the two groups. The cross-validated results indicated that these classifiers could not distinguish between the HC and MDD groups (Supplementary Fig. 4). Accordingly, within the present sample the estimated parameters did not exhibit any systematic group‑level differences between HC and MDD participants.

We further examined group differences in $E_{\text{ij}}$ (i.e., $E_{j}=w_{o}+w_{t}\;t_{j}+w_{a}\;a_{j}+\text{noise})$ between HC and patients with MDD. Across all photographed partners, participants with MDD consistently exhibited lower $E_{\text{ij}}$ values compared to HCs. The group differences in $E_{\text{ij}}$ were statistically significant for 18 out of the 41 photographed partners (Figure 5E and F). These findings are consistent with previous reports that individuals with MDD show reduced trust towards others [17], supporting the interpretation that $E_{\text{ij}}$ captures trust-related evaluation.

### Uncovering key biomarkers associated with decision-making in trusting behaviors

**Figure 6.**
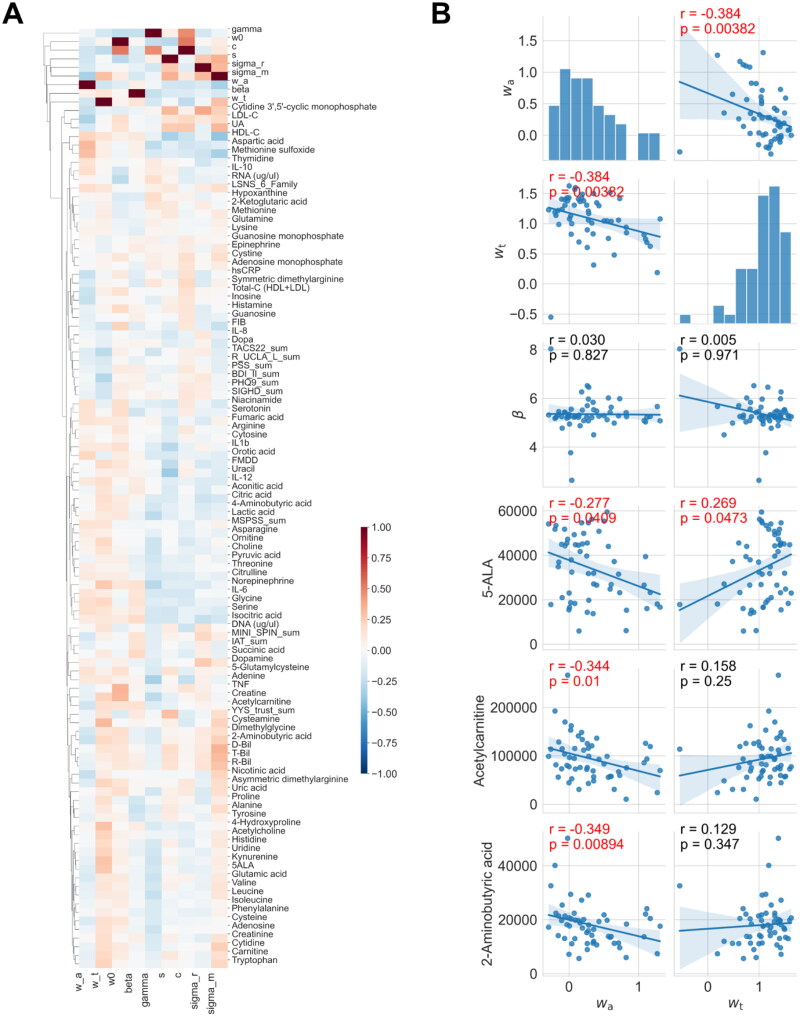
Screening of blood biomarkers correlated with the decision-making properties. **(A)** Heatmap of Pearson correlation coefficients between all estimated parameters and all measured blood metabolites. Hierarchical clustering was performed. Solid lines and shaded regions represent the estimated means and their 95% confidence intervals based on simple regression analysis, respectively. **(B)** Scatter plot of parameters and biomarkers for selected pairs. Correlation coefficients with an FDR-corrected q-value < 0.05 were considered significant, which were coloured by red.

To uncover hidden links between decision-making characteristics and metabolic states, we conducted a correlation analysis between participant-specific parameters and biomarkers (Figure 6A). By summarising the correlations (Figure 6A), we noticed some biomarkers correlated with participant-specific parameters estimated from trusting behaviours (q value = 0.05, false discovery rate), although most of them were not correlated with trusting behaviours (Figure 2A). We then identified 5ALA, acetyl carnitine, and 2-aminobutyric acid, which differed from the identified biomarkers that correlated with the participants’ answers and behaviours. These four biomarkers negatively correlated with wa, a weighting parameter for attractiveness (Figure 6B). 2-aminobutyric acid has been reported as a biomarker of depression [18]. Acetylcarnitine is related to oxidative stress [19], and several studies have reported both positive and negative correlations with depression [20,21]. Interestingly, acetyl carnitine is involved in the same metabolic pathway as acylcarnitine in the mitochondria, which we previously reported to be related to hikikomori [6]. There are no reports on the function of 5ALA in neurons and depression.

## Discussion

In this study, we performed a trusting game to obtain behavioural data on patients with MDD and healthy controls and measured metabolites from their blood samples. To simulate the trusting behaviour of each participant, we developed a Bayesian hierarchical model. By estimating the model parameters for each participant, we identified several metabolites as biomarkers that correlated with the estimated parameters of the decision-making model. Our findings suggest that our approach helps uncover hidden links between biological states and decision-making processes underlying trusting behaviour, providing new insights into the biological basis of trust.

### Trusting behaviours in psychiatric disorders and hikikomori

We hypothesised that patients with psychiatric disorders might have a weak level of trust in their surroundings and adopted a trust game to assess their trust-based behaviours. The psychiatric patients partially co-exhibit symptoms of "withdrawal", a lack of contact with the outside world. Therefore, modelling trusting behaviours is important to evaluate the decision-making process quantitatively.

In this study, some participants repeated the same answers and investments (Figure 4). Such biased outcomes reflect the fact that the participants refused to think and provide valid answers. By evaluating the biases based on Shannon’s information entropy (Figure 4), we found that patients with MDD had biased answers and investments compared with HCs, which is consistent with reports that patients with MDD exhibit autistic behaviour [22]. Thus, participants with such biases provided less information for further analysis of their trusting behaviours.

### Biological function of the identified biomarkers

In this study, we proposed three levels of biomarkers: those discriminating psychiatric states, those predicting participants’ behaviours (i.e., answers and investments), and those associated with decision-making processes.

Previously, we identified several biomarkers for discriminating between patients with MDD and HCs at the first biomarker level. For example, increased levels of C-reactive protein (CRP), interleukin-6 (IL-6), and tumour necrosis factor-alpha (TNF-α), and decreased levels of brain-derived neurotrophic factor (BDNF) and serotonin, have been found to differentiate between patients with MDD and HCs [23–26].

Herein, we identified several biomarkers that were highly correlated with behaviour (i.e., answers and investment) as secondary biomarkers. For example, participants with higher concentrations of uric acid, carnitine, methionine, creatinine, and nicotinic acid scored higher on the trustworthiness and attractiveness of the photographed partners and invested more in their partners (Figure 2). Although we did not measure oxytocin (OT) in blood, it is known to promote perceived facial trustworthiness and attractiveness [27]. However, such biomarkers, including OT, do not reflect how the subject made decisions based on external inputs, that is, the transformation from the facial evaluation of partners to investment.

At the third level of biomarkers, we identified several biomarkers in the blood associated with DMP. Acetylcarnitine is associated with the extent of attractiveness and oxidative stress. However, as it is well known, the concentration of metabolites in blood varies dramatically and can be influenced by numerous objective factors, such as sleep and diet. Therefore, further research is needed to determine how changes in acetylcarnitine concentrations affect changes in brain activity.

### Comparison with previous studies

Previous studies using fMRI have examined the neural basis of trusting behaviours and shown that trusting behaviours are associated with the activities of specific brain areas, such as the medial prefrontal cortex (mPFC) and temporo-parietal junction (TPJ) [28,29]. In social implementations, it is impractical to use expensive fMRI to assess trust behaviour. Our study aimed to use blood biomarkers to predict decision-making traits and trusting behaviours of participants. The biomarkers identified in this study enabled us to characterise DMP in a cost-effective manner.

There are several mathematical modelling studies on decision making in the trust game, compared to our model, which has the following advantages [30–34]: First, unlike models in previous studies, our model considered the subjective perception of a partner’s photograph, that is, the attractiveness and trustworthiness of the partner. Generally, mathematical modelling in a trust game rarely focuses on the effect of subjective perceptions on decision making. On the other hand, in our daily life, our trust behaviours are largely influenced by the appearance of partners. Therefore, our mathematical model can assess the reality of trust decisions. Second, we solved the inverse problem, that is, the estimation of the model parameters from the behavioural data for each participant in a personalised manner. All previous studies developed forward models that needed to be simulated using manually assigned parameters.

Thus, this study had the advantage of quantitatively assessing personality traits. Third, our modelling approach relates trusting decisions to biological mechanisms. Many studies on biomarkers and mathematical models have been conducted independently; however, our study integrated them. As the model parameters can be intuitively interpreted as decision-making properties, we can examine which blood metabolites correlate with the decision-making properties.

### Future perspective

Contrary to our expectations, we did not observe clear separability between the parameter sets of the HC and MDD groups. This pattern suggests that the nine participant-specific parameters may primarily reflect individual traits—such as personality—rather than diagnostic status. However, a definitive conclusion will require longitudinal data; specifically, re-estimating the same parameters for the same individuals across multiple sessions to evaluate their intra-individual stability over time.

Looking ahead, deep generative models could help refine the experimental design. Recent advances in Generative Adversarial Networks (GANs) allow facial images to be synthesised with precisely controlled levels of perceived trustworthiness and attractiveness [35]. Deploying such stimuli would minimise the subjective‑rating bias currently inherent in our model inputs. In addition, the present framework—focused on a one‑shot trust game—can be extended to iterated interactions. For example, modelling how the outcome of a previous round (e.g., being betrayed) modulates subsequent investment decisions, and whether this dynamic differs between MDD patients and HCs, represents an important next step.

## Methods

### Ethics statement

This case-control study was approved by the Ethics Committee of Kyushu University (30–452) and was conducted in accordance with the Declaration of Helsinki. The recruitment period for this study was from 25 June 2013, to 31 March 2021, and written informed consent was obtained. The original data used in this study were previously reported in [36], which includes datasets from earlier studies [3] and [12], along with additional data obtained from subsequent experiments conducted under the same protocol.

### Participants

We enrolled drug-free patients with MDD and HCs (age- and sex-matched controls) in the present study. All participants were Japanese (Asian) and provided written informed consent prior to the study. Patients were recruited from Kyushu University Hospital and its affiliations (mainly outpatient clinics). HCs were recruited *via* flyers at Kyushu University Hospital and the university campus. Structured Clinical Interview for DSM-IV-TR (SCID) was conducted by trained psychiatrists to diagnose MDD. We selected 38 drug-free patients with MDD (22 men and 16 women). As exclusion criteria for patients with MDD, we confirmed that none of the patients had a history of neurodegenerative diseases, psychotic disorders, mental retardation, substance abuse, or physical diseases, such as cardiovascular diseases, liver and kidney diseases, infectious diseases, malignant tumours, or head trauma. We selected 38 healthy participants as HCs through interviews based on the SCID regarding any previous or ongoing psychiatric disorders, physical diseases, and medications, which were set as the exclusion criteria.

### PC-based trust game

As introduced in our previous reports [3,12], we developed an original PC-based trust game, which was conducted using a laptop to evaluate participants’ trusting behaviours and preferences for others. The participants (trustors) were instructed on the game rules. Subsequently, they were required to make decisions regarding the amount of 1,300 JPY (about 12 USD) to give to each of the 40 partners (trustees) and to rate the partners’ attractiveness based on facial photographs presented on a computer screen (score ranges: 0–9), which is thought to reflect the raters’ subjective preference for others (Preference Score). The amount of money given to the partner by the participant (Monetary Score) is tripled, and the partner then decides whether to split the money equally with the participant or take the entire amount. The participant’s decision on the amount of money to give to the partner is thought to reflect their level of trust in the partner.

In the experiment, the partners were virtual players on a computer screen. The participants had no information about their partners except for facial photographs, including the head and shoulders, with a neutral facial expression. Photos of the partners were selected from professional fashion models (i.e., “high-attractive partners”) or lay individuals (i.e., “ordinary-attractive partners”). We randomly selected 40 pictures (10 each of professional male fashion models, professional female fashion models, lay males, and lay females) for the trust game. Hence, the faces of the photographed partners had different levels of attractiveness. Participants were unaware of their partners’ decisions. After the experiments, each participant was paid an amount of money corresponding to the result of a randomly selected game as a reward.

### Blood biomarkers

All peripheral venous blood samples were collected between 10:00 and 15:00. Plasma was immediately extracted, frozen, and stored at -80 °C until analysis. The measured blood biomarkers included routine blood biochemical markers and metabolites (metabolomics).

Routine blood biochemical markers including serum total-cholesterol (Total-C), high density lipoprotein-cholesterol (HDL-C), low density lipoprotein-cholesterol (LDL-C), fibrinogen (Fib), fibrin/fibrinogen degradation products (FDP), total-bilirubin (T-bil), direct-bilirubin (D-bil), indirect-bilirubin (I-bil), uric acid (UA), and high-sensitivity C-reactive protein (hsCRP) were measured by automatic biochemical analyser (SRL, Inc., Tokyo, Japan). Plasma metabolites were measured by liquid chromatography-mass spectrometry (LC-MS) using LCMS-8060 (Shimadzu Corp., Kyoto, Japan), as previously described [37].

### Generative model for trusting behaviours

Our Bayesian hierarchical model assumes that the evaluation of the *j-*th photographed partner by the *i*-th subject, $E_{i,j}$, is determined by the weighting of trustworthiness and attractiveness as follows:

$$E_{i,j}=w_{i}^{T}r_{i,j}+\sigma_{e\;i}\xi_{e\;i,j}$$

where $r_{i,j}=(a_{i,j},t_{i,j},1{)}^{T}$, $a_{i,j}$, and $t_{i,j}$ indicate the i-th participant’s rating of trustworthiness and attractiveness for the *j*-th photographed partners, respectively, and 1 represents common input among all partners; $w_{i}=(w_{a\;i},w_{t\;i},w_{o\;i}{)}^{T}$ indicates the weight vector for attractiveness, trustworthiness, and common input; $\xi_{e\;i,j}$ and $\sigma_{e\;i}$ indicate Gauss noise with zero mean and unit variance and its noise strength, respectively. Thus, $E_{i,j}$ obeys the Gaussian distribution

$$P\left(r_{i,j},w_{i},\sigma_{e,i}\right)=N\left(E_{i,j}w_{i}^{T}r_{i,j},\text{diag}\left(\sigma_{e,i}{}^{2}\right)\right)$$

where N(x|μ,Σ) represents a Gaussian distribution with mean μ and variance Σ; $\text{diag}(q_{i})$ represents a diagonal matrix whose *i*-th diagonal entry is $q_{i}$. The participants probabilistically decide whether to invest in a Bernoulli distribution.

$$P(E_{i,j},\beta_{i},\gamma_{i})=\mu (E_{i,j}{)}^{v}(1-\mu (E_{i,j}){)}^{\left(1-v\right)}$$

where *v* indicates binary variables; that is, 1 and 0 correspond to investment and no investment, respectively. $\mu (E_{i,j})$ is sigmoidal function:

$$\mu (E_{i,j})=\frac{1}{\left(1+\text{exp}(-\beta_{i}(E_{i,j}-\gamma_{i}))\right)}$$

where $\beta_{i}$ and $\gamma_{i}$ indicate participant-specific constants that control for randomness and threshold of investment, respectively. If the investment is decided, the participant decides the amount of money as:

$$m_{i,j}=s_{i}E_{i,j}+c_{i}+\sigma_{m,i}\xi_{m\;j}$$

where $s_{i}$ and $c_{i}$ are participant-specific constants. If the participant does not select an investment, the amount of money is zero; thus, $m_{i,j}$ obeys the following distribution:

$$P(v_{i,j},E_{i,j},s_{i},c_{i},\sigma_{m,i})=N(m|s_{i}E_{i,j}+c_{i},{\sigma_{m,i}}^{2}{)}^{v_{i,j}}*\delta (m_{i,j}{)}^{\left(1-v_{i,j}\right)}$$

where δ(x) indicates the delta function where δ(x=0)=1 and δ(x≠0)=0.

### Prior distributions

For parameter estimation in a Bayesian manner, we introduced the following prior distributions:

$$P(w_{a})=N(w_{a0},\sigma_{w_{a0}})$$

$$P(w_{t})=N(w_{t0},\sigma_{w_{t0}})$$

$$P(w_{o})=N(w_{o0},\sigma_{w_{o0}})$$

$$P(\beta )=\text{Gamma}(\text{shap}e_{\beta 0},{\text{scale}}_{\beta 0})$$

$$P(\gamma )=N(\gamma_{o},\sigma_{\gamma 0})$$

$$P(\sigma_{e})=N_{+}(\sigma_{e0})$$

$$P(s)=N(s_{0},\sigma_{s0})$$

$$P(c)=N(c_{0},\sigma_{c0})$$

$$P(\sigma_{m})=N_{+}(\sigma_{m0})$$

where $N(x|\mu ,\sigma^{2})$ denotes a normal distribution with mu as the mean and sigma as the variance, Gamma(a,b) indicates a Gamma distribution with shape parameter *a* and rate parameter *b, and* $N_{+}(\sigma )$ indicates a half-normal distribution with scale σ. For most parameters, we used a normal distribution as the prior distribution. Because of the need to restrict beta to positive values, we chose the gamma distribution as the prior distribution. For the prior distribution of the variance parameter, we used a commonly used half-normal distribution of the variance parameter.

In the MCMC, we used $w_{a0}{=w}_{t0}=w_{o0}=0$, $\sigma_{w_{a0}}=\sigma_{w_{t0}}=\sigma_{w_{o0}}=1$, $\text{shap}e_{\beta 0}=5$, ${\text{scale}}_{\beta 0}=1$, $\gamma_{o}=\sigma_{\gamma 0}=1$, $\sigma_{e0}=0.5$, $s_{0}=\sigma_{s0}=c_{0}=\sigma_{c0}=100$, and $\sigma_{m0}=100$.

### Bayesian inference of parameters

We computed the posterior distribution of the parameters as

$$\begin{array}{l} P(\theta_{i}a_{i,j},t_{i,j},v_{i,j},m_{i,j})\propto P(E_{i,j}a_{i,j},t_{i,j},w_{o\;i},w_{a\;i},w_{t\;i},\sigma_{\text{ei}})\\ P(v_{i,j}E_{i,j},\beta_{i},\gamma_{i})P(m_{i,j}v_{i,j},E_{i,j},s_{i},c_{i},\sigma_{\text{mi}})P\left(\theta_{i}\right) \end{array}$$

where $\theta_{i}=\left\{w_{o\;i},w_{a\;i},w_{t\;i},\sigma_{e\;i},\sigma_{m\;i},\beta_{i},\gamma_{i,}s_{i},c_{i}\right\}$ and $P(\theta_{i})$ indicate prior distribution as $P(w_{o\;i})P(w_{a\;i})P(w_{t\;i})P(\sigma_{e\;i})P(\beta_{i})P(\gamma_{i})P(s_{i})P(c_{i})P(\sigma_{m\;i})$. The inference of this distribution cannot be computed analytically; therefore, we employed Markov Chain Monte Carlo (MCMC) method to perform the estimation. We used the No-U-turn sampler (NUTS) to sample this distribution, where 2,000 samples were drawn for burn-in and another 4,000 samples were drawn to estimate the distribution. MCMC was used to obtain point estimates of individual parameters, approximating the maximum a posteriori (MAP) estimates.

### Cross-validation

In the trust game, participants rated and invested in 41 partners. For each participant, we optimised the model using the ratings and investment amounts of 31 partners as the training set and obtained their specific parameters. In the participant-specific model, we predicted the investment and monetary scores of the remaining ten partners. We calculated the regression coefficient r^2 to estimate the credibility of the model.

## Supplementary Material

SupplementFig_20250522.docx

## Data Availability

The original data used in the present study were previously reported [36] and are available on GitHub at https://github.com/ZMCao37/trustgameModel.git.

## Code availability statement

The data analysis was performed using Jupyter Lab. The code used for this work is available on GitHub at https://github.com/ZMCao37/trustgameModel.git.

## References

[1] Lee IC, Chen EE, Yen NS, Tsai CH, Cheng HP. 2017. Are we rational or not? The exploration of voter choices during the 2016 presidential and legislative elections in Taiwan. Front Psychol. 8:1762. doi:10.3389/fpsyg.2017.01762. 29075215 PMC5643908

[2] Greenwood M, van Buren HJ. 2010. Trust and stakeholder theory: trustworthiness in the organisation-stakeholder relationship. J Bus Ethics. 95(3):425–438. doi:10.1007/s10551-010-0414-4.

[3] Watabe M, Kato TA, Teo AR, Horikawa H, Tateno M, Hayakawa K, Shimokawa N, Kanba S. 2015. Relationship between trusting behaviors and psychometrics associated with social network and depression among young generation: a pilot study. PLoS One. 10(3):e0120183. doi:10.1371/journal.pone.0120183. 25836972 PMC4383339

[4] Kato TA, Kanba S, Teo AR. 2019. Hikikomori: multidimensional understanding, assessment and future international perspectives. Psychiatry Clin Neurosci. 73(8):427–440. pcn doi:10.1111/pcn.12895. 31148350

[5] Kato TA, Katsuki R, Kubo H, Shimokawa N, Sato‐Kasai M, Hayakawa K, Kuwano N, Umene‐Nakano W, Tateno M, Setoyama D, et al. 2019. Development and validation of the 22‐item Tarumi’s Modern‐Type Depression Trait Scale: avoidance of Social Roles, Complaint, and Low Self‐Esteem (TACS‐22). Psychiatry Clin Neurosci. 73(8):448–457. doi:10.1111/pcn.12842. 30900331 PMC6850625

[6] Setoyama D, Matsushima T, Hayakawa K, Nakao T, Kanba S, Kang D, Kato TA. 2021. Blood metabolic signatures of hikikomori, pathological social withdrawal. Dialogues Clin Neurosci. 23(1):14–28. doi:10.1080/19585969.2022.2046978. 35860171 PMC9286746

[7] Ellis AJ, Vanderlind WM, Beevers CG. 2013. Enhanced anger reactivity and reduced distress tolerance in major depressive disorder. Cogn Ther Res. 37(3):498–509. doi:10.1007/s10608-012-9494-z.

[8] Moutoussis M, Rutledge RB, Prabhu G, Hrynkiewicz L, Lam J, Ousdal O-T, Guitart-Masip M, Fonagy P, Dolan RJ. 2018. Neural activity and fundamental learning, motivated by monetary loss and reward, are intact in mild to moderate major depressive disorder. PLoS One. 13(8):e0201451. doi:10.1371/journal.pone.0201451. 30071076 PMC6072018

[9] Tzieropoulos H. 2013. The Trust Game in neuroscience: a short review. Soc Neurosci. 8(5):407–416. doi:10.1080/17470919.2013.832375. 23998424

[10] Preuss N, Brändle LS, Hager OM, Haynes M, Fischbacher U, Hasler G. 2016. Inconsistency and social decision making in patients with Borderline Personality Disorder. Psychiatry Res. 243:115–122. doi:10.1016/j.psychres.2016.06.017. 27380424

[11] Seres I, Unoka Z, Kéri S. 2009. The broken trust and cooperation in borderline personality disorder. Neuroreport. 20(4):388–392. doi:10.1097/WNR.0b013e328324eb4d. 19218873

[12] Hayakawa K, Kato TA, Watabe M, Teo AR, Horikawa H, Kuwano N, Shimokawa N, Sato-Kasai M, Kubo H, Ohgidani M, et al. 2018. Blood biomarkers of Hikikomori, a severe social withdrawal syndrome. Sci Rep. 8(1):2884. doi:10.1038/s41598-018-21260-w. 29440704 PMC5811600

[13] Kishi T, Yoshimura R, Ikuta T, Iwata N. 2018. Brain-derived neurotrophic factor and major depressive disorder: evidence from meta-analyses. Front Psychiatry. 8:308. doi:10.3389/fpsyt.2017.00308. 29387021 PMC5776079

[14] Wysokiński A, Margulska A, Strzelecki D, Kłoszewska I. 2015. Levels of C-reactive protein (CRP) in patients with schizophrenia, unipolar depression and bipolar disorder. Nord J Psychiatry. 69(5):346–353. doi:10.3109/08039488.2014.984755. 25495587

[15] Kato TA, Watabe M, Tsuboi S, Ishikawa K, Hashiya K, Monji A, Utsumi H, Kanba S. 2012. Minocycline modulates human social decision-making: possible impact of microglia on personality-oriented social behaviors. PLoS One. 7(7):e40461. doi:10.1371/journal.pone.0040461. 22808165 PMC3396661

[16] Shannon CE. 1948. A Mathematical Theory of Communication. Bell System Technical Journal. 27(3):379–423. doi:10.1002/j.1538-7305.1948.tb01338.x.

[17] Wang T, Zeng J, Peng P, Yin Q. 2024. Social decision-making in major depressive disorder: a three-level meta-analysis. J Psychiatr Res. 176:293–303. doi:10.1016/j.jpsychires.2024.06.026. 38905762

[18] Adachi Y, Toyoshima K, Nishimoto R, Ueno S, Tanaka T, Imaizumi A, Arashida N, Nakamura M, Abe Y, Hakamada T, et al. 2019. Association between plasma α-aminobutyric acid and depressive symptoms in older community-dwelling adults in Japan. Geriatr Gerontol Int. 19(3):254–258. doi:10.1111/ggi.13585. 30561103

[19] Liu J, Head E, Kuratsune H, Cotman CW, Ames BN. 2004. Comparison of the effects of L-carnitine and acetyl-L-carnitine on carnitine levels, ambulatory activity, and oxidative stress biomarkers in the brain of old rats. Ann N Y Acad Sci. 1033:117–131. doi:10.1196/annals.1320.011. 15591009

[20] Pettegrew JW, Levine J, Mcclure RJ. 2000. Acetyl-L-carnitine physical-chemical, metabolic, and therapeutic properties: relevance for its mode of action in Alzheimer’s disease and geriatric depression. 5. Mol Psychiatry. 5(6):616–32. doi:10.1038/sj.mp.4000805. 11126392

[21] Nasca C, Bigio B, Lee FS, Young SP, Kautz MM, Albright A, Beasley J, Millington DS, Mathé AA, Kocsis JH, et al. 2018. Acetyl-L-carnitine deficiency in patients with major depressive disorder. Proc Natl Acad Sci USA. 115(34):8627–8632. doi:10.1073/pnas.1801609115. 30061399 PMC6112703

[22] Naguy A. 2021. Major depressive disorder in autism spectrum disorder. Prim Care Companion CNS Disord. 23(6):95. doi:10.4088/PCC.20br02895. 34710953

[23] Dowlati Y, Herrmann N, Swardfager W, Liu H, Sham L, Reim EK, Lanctôt KL. 2010. A meta-analysis of cytokines in major depression. Biol Psychiatry. 67(5):446–457. doi:10.1016/j.biopsych.2009.09.033. 20015486

[24] Haapakoski R, Mathieu J, Ebmeier KP, Alenius H, Kivimäki M. 2015. Cumulative meta-analysis of interleukins 6 and 1β, tumour necrosis factor α and C-reactive protein in patients with major depressive disorder. Brain Behav Immun. 49:206–215. doi:10.1016/j.bbi.2015.06.001. 26065825 PMC4566946

[25] Sen S, Duman R, Sanacora G. 2008. Serum brain-derived neurotrophic factor, depression, and antidepressant medications: meta-analyses and implications. Biol Psychiatry. 64(6):527–532. doi:10.1016/j.biopsych.2008.05.005. 18571629 PMC2597158

[26] Owens MJ, Nemeroff CB. 1994. Role of serotonin in the pathophysiology of depression: focus on the serotonin transporter. Clin Chem. 40(2):288–295. doi:10.1093/clinchem/40.2.288. 7508830

[27] Kosfeld M, Heinrichs M, Zak PJ, Fischbacher U, Fehr E. 2005. Oxytocin increases trust in humans. Nature. 435(7042):673–676. doi:10.1038/nature03701. 15931222

[28] McCabe K, Houser D, Ryan L, Smith V, Trouard T. 2001. A functional imaging study of cooperation in two-person reciprocal exchange. Proc Natl Acad Sci USA. 98(20) 11832–11835. doi:10.1073/pnas.211415698. PMC58817 11562505

[29] Alós-Ferrer C. 2018. A review essay on social neuroscience: can research on the social brain and economics inform each other? J Econ Lit. 56(1):234–264. doi:10.1257/jel.20171370.

[30] Xiang T, Ray D, Lohrenz T, Dayan P, Montague PR. 2012. Computational phenotyping of two-person interactions reveals differential neural response to depth-of-thought. PLoS Comput Biol. 8(12):e1002841. doi:10.1371/journal.pcbi.1002841. 23300423 PMC3531325

[31] Peixoto TP, Bornholdt S. 2012. No need for conspiracy: self-organized cartel formation in a modified trust game. Phys Rev Lett. 108(21):218702. doi:10.1103/PhysRevLett.108.218702. 23003311

[32] Masuda N, Nakamura M. 2012. Coevolution of trustful buyers and cooperative sellers in the trust game. PLoS One. 7(9):e44169. doi:10.1371/journal.pone.0044169. 22970176 PMC3436922

[33] Manapat ML, Nowak MA, Rand DG. 2013. Information, irrationality, and the evolution of trust. J Econ Behav Organ. 90:S57–S75. doi:10.1016/j.jebo.2012.10.018.

[34] Chang LJ, Doll BB, van ‘t Wout M, Frank MJ, Sanfey AG. 2010. Seeing is believing: trustworthiness as a dynamic belief. Cogn Psychol. 61(2):87–105. doi:10.1016/j.cogpsych.2010.03.001. 20553763

[35] Nightingale SJ, Farid H. 2022. AI-synthesized faces are indistinguishable from real faces and more trustworthy. Proc Natl Acad Sci USA. 119(8):19. doi:10.1073/pnas.2120481119. PMC8872790 35165187

[36] Kubo H, Setoyama D, Watabe M, Ohgidani M, Hayakawa K, Kuwano N, Sato-Kasai M, Katsuki R, Kanba S, Kang D, et al. 2021. Plasma acetylcholine and nicotinic acid are correlated with focused preference for photographed females in depressed males: an economic game study. Sci Rep. 11(1):2199. doi:10.1038/s41598-020-75115-4. 33500434 PMC7838250

[37] Kuwano N, Kato TA, Setoyama D, Sato-Kasai M, Shimokawa N, Hayakawa K, Ohgidani M, Sagata N, Kubo H, Kishimoto J, et al. 2018. Tryptophan-kynurenine and lipid related metabolites as blood biomarkers for first-episode drug-naïve patients with major depressive disorder: an exploratory pilot case-control study. J Affect Disord. 231:74–82. doi:10.1016/j.jad.2018.01.014. 29454180

