# Supplementary material for: Leveraging machine learning to uncover the hidden links between trusting behavior and biological markers
Source: Dialogues Clin Neurosci. 2025 Jun 20;27(1):201–15. doi: 10.1080/19585969.2025.2513697 (PMC12897530; doi:10.1080/19585969.2025.2513697)
Supplement: SupplementFig_20250522.docx [file TDCN_A_2513697_SM9165.docx]

**
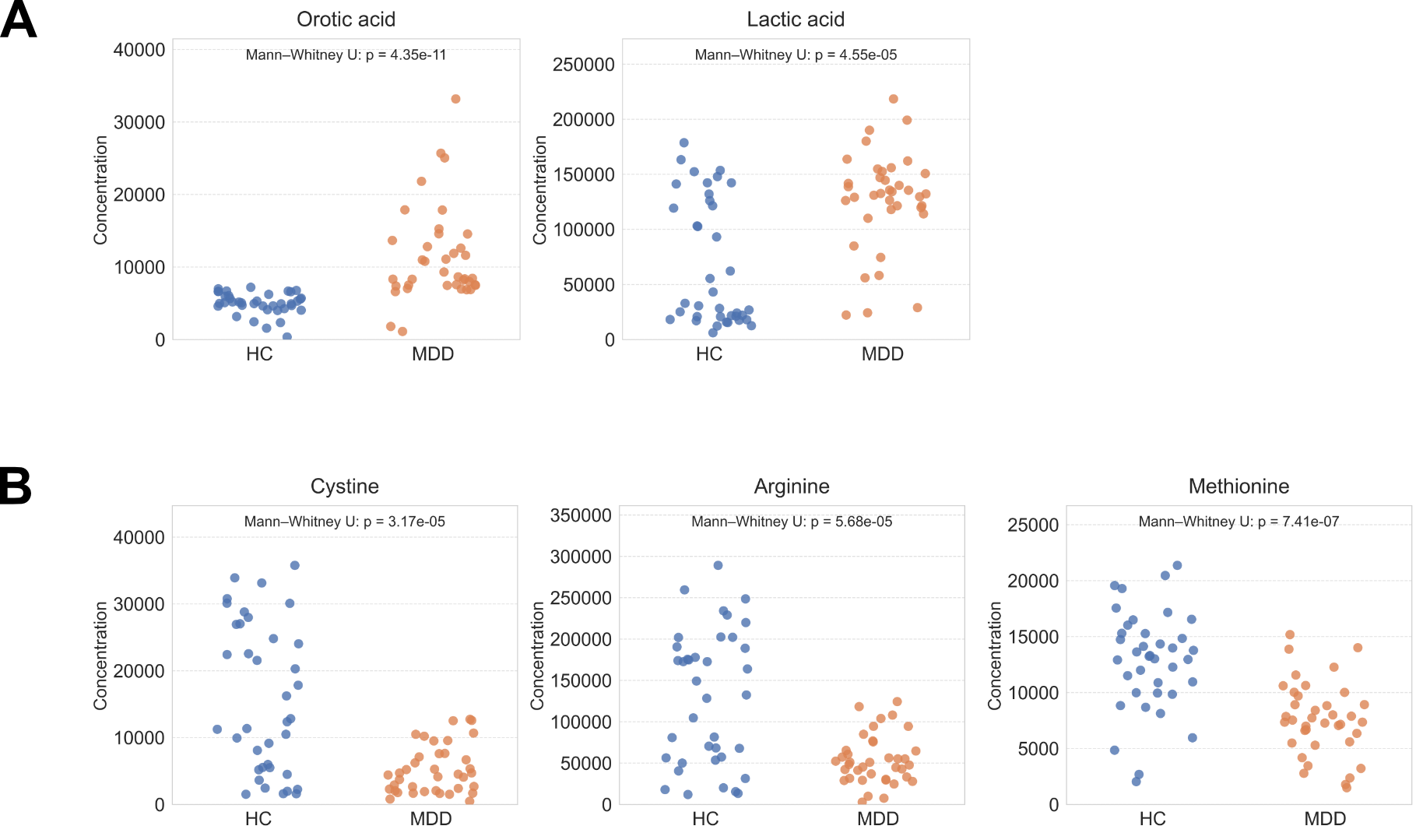
**

**Supplementary Figure 1: Comparison of blood biomarkers that differ significantly between the MDD and HC groups**(A) Biomarkers with elevated plasma levels in patients with MDD (orotic acid and lactic acid). (B) Biomarkers with reduced plasma levels in patients with MDD (cystine, arginine, and methionine). Group differences were assessed using the Mann–Whitney U test.


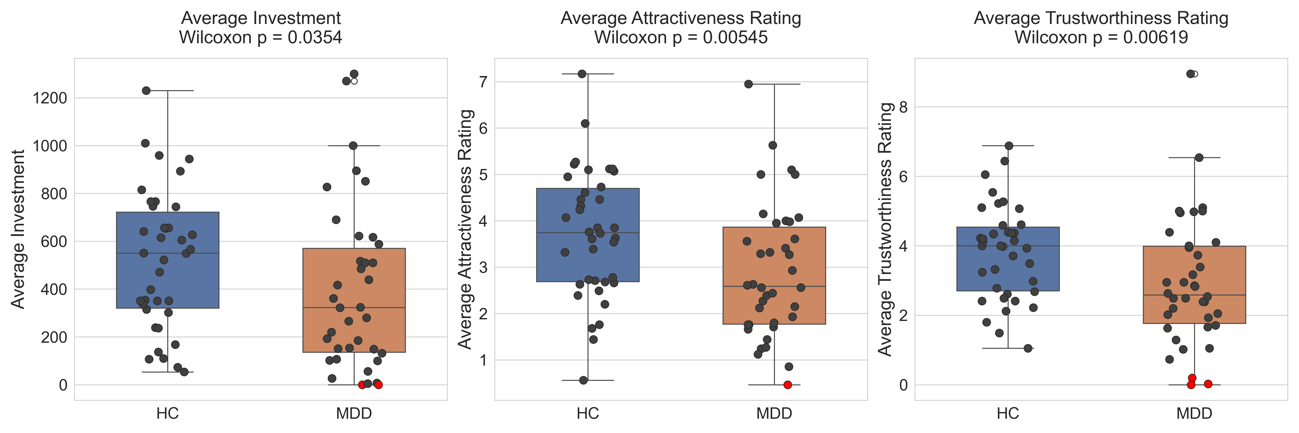


**Supplementary Figure 2: Comparison of behavioral responses between HCs and patients with MDD**Distribution of individual participants’ average values for investment amounts (left), attractiveness ratings (center), and trustworthiness ratings (right) across all photographed partners. Each dot represents one participant. Statistical testing was conducted using the Wilcoxon rank-sum test. Error bars represent the standard deviation.


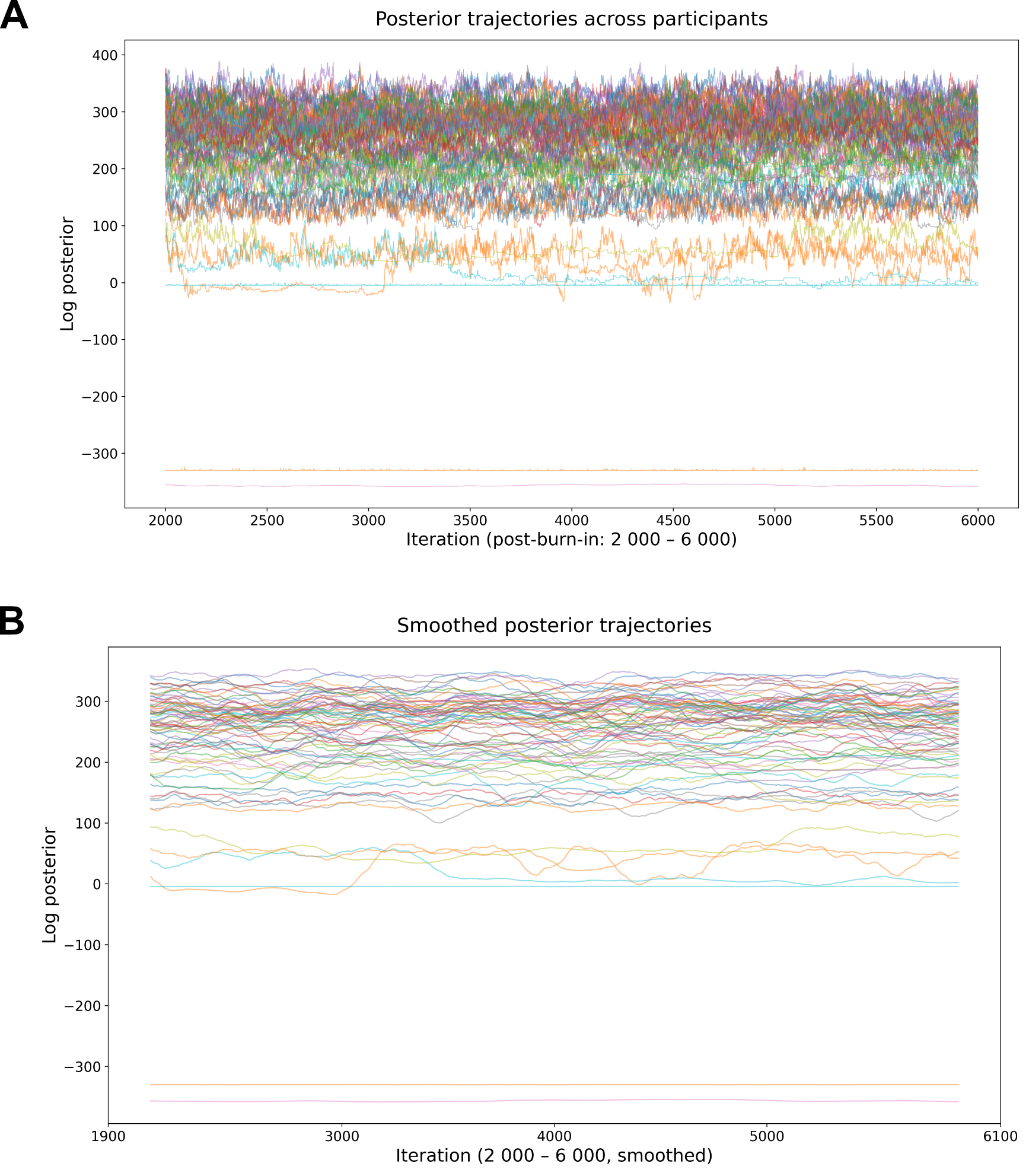


**Supplementary Figure 3: Convergence check of the MCMC inference**(A) Trajectories of the maximum posterior values over 6,000 NUTS iterations. (B) The same trajectories smoothed with a moving average (window = 200 iterations). Because NumPyro discards the first 2000 burn-in iterations, only the 4000 sampling iterations are displayed.

**
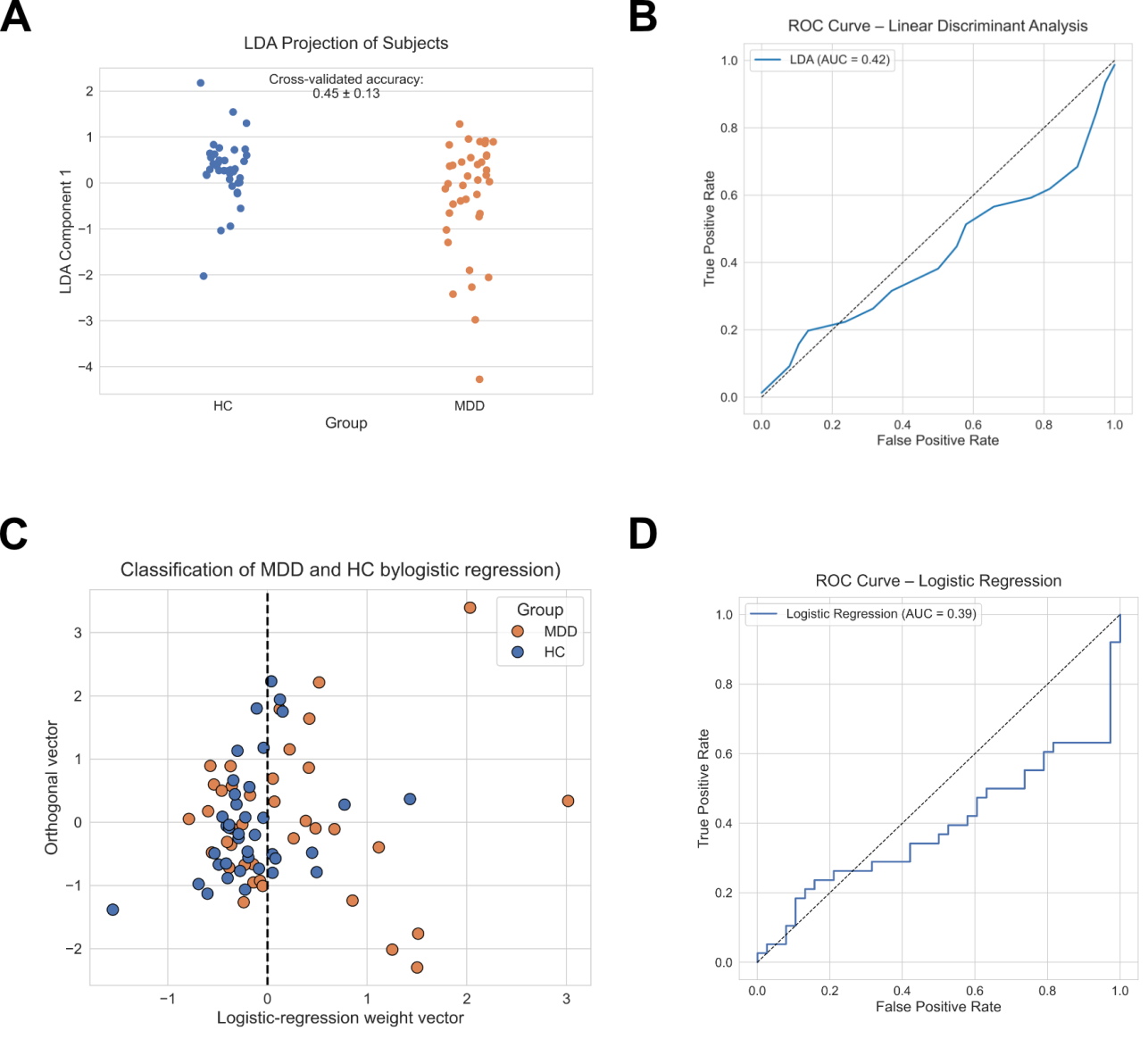
Supplementary Figure 4: No clear separability between MDD and HC groups by supervised classification**

(A) Classification of MDD and HC groups based on the estimated model parameters using Linear Discriminant Analysis (LDA). Each dot represents the value of the first linear discriminant axis projected from a participant’s model parameters. The cross-validated classification accuracy (5-fold cross-validation) is indicated in the panel. (B) Receiver operating characteristic (ROC) curve for the LDA classifier shown in (A). (C) Classification of MDD and HC groups based on the estimated model parameters using logistic regression. Each dot represents a participant projected onto the weight vector of the logistic regression model (x-axis) and its orthogonal vector (y-axis). The decision boundary (x = 0) is overlaid. (D) ROC curve for the logistic regression classifier shown in (C).
